# Applications of advanced in vitro ocular models

**DOI:** 10.1007/s44164-026-00113-1

**Published:** 2026-04-16

**Authors:** Barend J Groenewald, Marguerite Blignaut, Ben Loos, Nireshni Chellan

**Affiliations:** 1 Biomedical Research and Innovation Platform, South African Medical Research Council, Tygerberg, Cape Town, South Africa; 2 Centre for Cardio-Metabolic Research in Africa, Division of Medical Physiology, Faculty of Medicine and Health Sciences, Stellenbosch University, Cape Town, South Africa; 3 Department of Physiological Sciences, Stellenbosch University, Stellenbosch, South Africa

**Keywords:** Organoids, Organ-on-a-chip, Tissue engineering, Ocular, In vitro, Eye, Clinical application

## Abstract

Advanced in vitro models are becoming increasingly popular over traditional monolayer cell culture models as they more closely recapitulate key morphological and biochemical aspects of tissue similar to what is found in vivo. Although it is primarily used in disease modelling and drug discovery, the use of these models is expanding into clinical applications as well, including in the field of advanced ocular in vitro models. The eye is a complex organ with multiple cell types and layers, all playing a unique role in visual acuity. Although no singular model is yet capable of recreating the entire complexity of the eye, there are multiple models available that can accurately mimic the tissue of interest. This review will first give a brief overview of the anatomy and function of the eye before focussing on the potential applications of advance in vitro models, with a focus on scaffold-free, scaffold-based, and microphysiological systems. We highlight the role of these models in both pre-clinical and clinical applications, showcasing the importance of these in vitro models.

## Introduction

Ocular pathologies leading to blindness and vision loss is on a rise due to the aging population [1]. This increase is becoming more and more prevalent in low to middle income countries which carries about 90% of the global burden [2]. This high burden of ocular diseases is usually due to lower accessibility and availability to ocular care services, as well as eye care literacy [3].

Animal models are often used in ocular research to better understand the pathologies of diseases [4]. Animals like mice have been used in translational studies for retinal development and degenerative diseases [5] and subretinal fibrosis [6]. Rabbits are also often used in ocular studies involving elucidating disease mechanisms for corneal endothelial dysfunction [7] as well as investigating treatment efficacies [8]. Unfortunately, the species differences between humans and animals, such as different cellular proliferative capacity [9], anatomical differences [10], and response to drugs [11], does hinder the translatability of these models. These models are also controversial and raise several ethical concerns highlighting the need for New Approach Methodologies (NAMs). The need for NAMs have further been highlighted by the United States Food and Drug Administration, who no longer require animal testing for certain aspects of pharmaceutical development [12].

Following the four R’s (reduce, replace, refine and responsibility) of animal research, in vitro cell culture models are often an alternative to animal models [13, 14]. In vitro cell culture models are very effective in studying cellular integrity, drug uptake and transport, as well as understanding disease pathology [15]. However, the traditional two-dimensional (2D) monolayer cell culture models are considered biologically less relevant and overly simplistic [16], therefore advanced three-dimensional (3D) models are becoming more prevalent and favoured. These models provide a more physiologically relevant 3D environment to conduct various studies including disease modelling, drug discovery, and personalised medicine [17, 18].

With the development of advanced 3D models, the field of in vitro ocular research has been reshaped. These 3D models recapitulate ocular biology better than 2D models [19]. This improves mechanistic studies, drug development, and disease research, but also opens the possibility of personalised medicine, as well as clinical applications for in vitro models. The use of patient specific cells within these models improves the direct translation of drug efficacy or disease progression to the clinical aspect [20]. Furthermore, these advanced models also allow the generation of tissue-like structures that can be directly used as transplant grafts or in cell replacement therapy [21].

Various 3D in vitro models are available, which for the purposes of this review are grouped into three main categories: scaffold-free (self-assembled) systems, scaffold-based systems, and microphysiological systems (MPS). Scaffold-free systems rely on the self-assembly nature of cells into aggregates such as spheroids or organoids without the need for external scaffolds [22], whereas scaffold-based systems require the addition of materials such as hydrogels to achieve a 3D nature [23]. Microphysiological systems differ from scaffold-based models due to the addition of external devices to maintain and support the cells in vitro, and include organ-on-a-chip and perfusable systems [24]. Models can also combine various techniques across these three categories to achieve the optimal model required, such as combining organoids and organ-on-a-chip technology [25].

This review will highlight the applications of advanced ocular in vitro models, currently limited to disease modelling, drug discovery, understanding cellular physiology, and pre-clinical as well as some limited clinical applications, as highlighted by the representative models discussed in this paper. We will give a brief overview of the anatomy of the eye, as well as common ocular pathologies within specific regions and/or structures to highlight the need for 3D models. An overview of the importance of three-dimensional models precedes a succinct review of specialised advanced cell culture models of the eye, to highlight the applications that these models have.

## Anatomy of the human eye

**Fig. 1:**
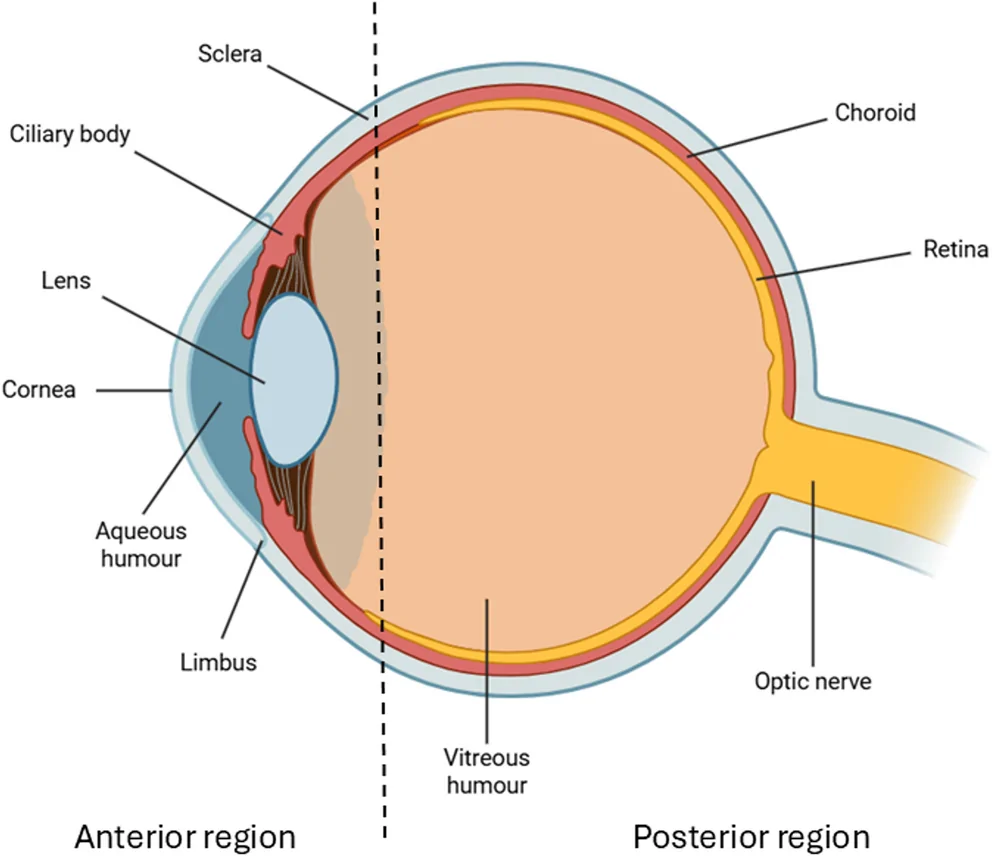
Cross-section of the eye. The eye can be divided into an anterior and posterior section separated by the lens. The anterior section contains majority of the structures and includes the cornea, sclera, ciliary body, and the lens. Meanwhile the retina makes up the posterior section, with accessory structures such as the optic nerve

The human eye is a highly complex structure that consists of numerous cell types, some of which are highly specialised. The eye is a fluid filled spheroidal structure that converts light into electrical signals to form images in the brain. It is made up of three main structures, namely the corneal-scleral, uveal, and retinal layers, moving anterior to posterior (Fig. 1).

The corneal-scleral layer of the eye forms the anterior aspect, and consists of a tough fibroelastic tissue that provides support for the rest of the eye. The anterior portion of this layer, the cornea, is a transparent structure that is the main refractive medium of the eye. The cornea is comprised of 5 distinct layers: the epithelium, Bowman’s membrane, stroma, Descemet’s membrane, and the endothelium as previously summarised [26]. Limbal stem cells (LSCs) residing in the Palisades of Vogt in the transitional region between the cornea and the conjunctiva known as the limbus, allows for a high epithelial turnover rate [27]. Single-cell RNA sequencing of over 21 000 cells from adult human corneas and conjunctiva identified a range of transcriptionally unique cell clusters. These clusters represent epithelial, endothelial, stromal, immune, vascular and melanocyte populations. Multiple specialised epithelial cell subpopulations such as basal, superficial, limbal progenitor cells, as well as keratocytes, fibroblasts and endothelial cells were also identified [28]. The sclera is a white fibrous tissue that provides insertion points for the extraocular muscles [29]. These biomechanical properties of the sclera are essential in its role to provide stability and support to the eye [30].

As previously reviewed, the uveal tract consists of the iris, ciliary body, and the choroid, and is responsible for focusing the light through the aperture, producing the internal fluids of the eye, as well as supplying and removing wastes and nutrients to the other layers respectively [31]. This layer is also immunologically active, containing numerous resident macrophages and dendritic cells throughout the uveal tract, and is well described by McMenamin (1997) [32]. The choroid represents the highly vascular posterior portion of the uveal tract and is located between the sclera and the retina. There are five layers that make up the choroid: Bruch’s membrane, the choriocapillaris, Sattler’s layer, Haller’s layer, and the suprachoroidal layer. These layers are composed of connective tissue and a wide range of cell types that include melanocytes, smooth muscle cells, choroidal neurons, mast cells, and pericytes [33].

The posterior part of the eye is the retina that consists of an inner neural layer and an outer retinal pigment epithelium (RPE). The neural layer of the retina can be divided into five distinct layers: the photoreceptor-containing outer nuclear layer, the inner nuclear layer, ganglion cell layer, outer plexiform layer, and an inner plexiform layer [34]. The nervous tissue layer contains photoreceptors known as cones and rods, which convert the light into neural signals, while the pigmented layer absorbs light and prevents any reflection or scattering of light within the eye [35]. Other cell types present in the neural retina as identified by single-cell RNA sequencing include bipolar cells, amacrine cells, retinal ganglion cells, retinal astrocytes, Müller glia, as well as microglia, endothelial cells, and pericytes [36]. The pigmented layer mainly consists of retinal pigment epithelial cells, enzymatically active cells that process retinoids required for phototransduction [37] and also maintain the photoreceptor outer segments through phagocytosis [38], prevent light scattering by absorbing excess light [39], protect against oxidative damage [40], and provide metabolic support to the photoreceptors [41].

All these layers and tissues are responsible for visual, and therefore any disruption or abnormalities can lead to vision impairment or loss. It is also clear that the eye is made up of numerous different cell types, resulting in critical cell-to-cell interactions which are an important consideration when trying to model the eye in vitro.

### Visual impairments

In 2020, an estimated 1.1 billion people suffered from either short or distant vision impairments, with 90% of those affected coming from low to middle income countries [42]. When looking at the age-standardised years lived with disability for blindness and vision loss between 1990 and 2019, Sub-Saharan Africa ranked amongst the highest [43]. The causes of vision loss or impairment can vary from genetic, lifestyle induced, or environmental causes [44].

Inflammation plays a major role in ocular diseases and can either be symptomatic or a causative agent in disease progression. The inflammatory disease bacterial keratitis often caused by *Pseudomonas aeruginosa*, is regarded as one of the leading causes for global ocular morbidity [45]. Inflammation is also prominent in dry eye disease, which is characterised by a disrupted tear film homeostasis resulting in surface inflammation and damage [46]. In the case of Stevens Johnson Syndrome (SJS) and toxic epidermal necrolysis (TEN) the acute inflammatory reaction of various mucosal membranes, including the ocular mucosa, is caused by an adverse reaction to drugs. Multi-ingredient, non-steroidal anti-inflammatories as well as cold medicines [47] are often the main causes of SJS and TEN. Moreover, SJS has a higher prevalence in HIV+ individuals placing a double burden on countries with high HIV-infection rates, like sub-Saharan Africa [48].

Genetic conditions are also common in ocular pathology. Leber’s congenital amaurosis (LCA), is one of the most severe forms of inherited retinal dystrophies, and accounts for 20% of visual impairments and blindness seen in children. Since the discovery of the first LCA-linked gene, *GUCY2D*, 19 other mutations in genes have been identified, and depending on the causative gene, patients can present a variety of symptoms [49]. Retinitis pigmentosa, a heterogeneous retinopathy that causes photoreceptor cell death and leads to blindness, is another well-known genetic abnormality Affecting about 1:5000 people world-wide, depending on the location of the causative gene, it can be inherited either autosomal-recessive, autosomal-dominant, or in a X-linked manner [50].

However, ocular pathologies are often a result of multiple factors. Limbal stem cell deficiency (LSCD) can be due to a genetic defect, or as a result of injury or damage to the limbal area. The corneoscleral limbus provides an optimal microenvironment for corneal epithelial stem cells, and any abnormalities to this region can cause abnormal corneal wound healing [51]. Pathologies like keratoconus can also alter the morphology of certain structures in the eye. Keratoconus is characterised by progressive thinning and cone like morphology of the cornea in a bilateral manner [52]. Lastly, other systemic pathologies can also lead to ocular complications, diabetic retinopathy being one of the best-known examples. The increased oxidative stress seen in diabetes mellitus, can lead to the development of vascular abnormalities within the retina, such as haemorrhages and microaneurysms, and is projected to affect 130 million individual worldwide in 2030 [53, 54].

It is therefore clear that the eye is a complex organ affected by multifactorial diseases. It is therefore critical to establish human-relevant models to study these complex structures and diseases in an ethical manner.

### The role of 2D in vitro models in ocular research

The commonly used Draize eye test is the best known animal model to test ocular side effects of chemicals by applying the test chemical to one eye of rabbits and monitoring for adverse reactions [55]. However, this is met with great ethical concern, and therefore in vitro NAMs are often used. Two-dimensional in vitro models are some of the most used models in ocular research due to their inexpensive nature and that these models are well established in literature, and therefore it is easy to compare models [56]. These models are still often used in toxicological, pharmacokinetic, and pharmacodynamic studies, in accordance with the four R’s of ethical research [57]. Looking at the use of human corneal in vitro models, these cell lines have been used to investigate corneal drug absorption [58, 59], toxicity testing [60–62], as well as in ocular transporter studies [63].

Cell lines used in in vitro models can broadly be classified as either primary cells or immortalised cell lines. Primary cells are isolated directly from tissue without altering the proliferation capacity of these cells. They maintain their phenotype in vitro but only for a limited time span [64]. Immortalised cell lines, however, can be maintained for a much longer period. Immortalised cell lines can either be derived from cancerous tissue that allows the cells to proliferate indefinitely [65], or the cells can be altered via the introduction of telomerase [66], or transfection with constructs altering the proliferation signalling of cells [67].

Numerous immortalised cell lines are commercially available, including human retinal pigment epithelial ARPE-19 [68], rat derived cone photoreceptor 661 W [69], human corneal epithelial HCE-T [70], and rat conjunctival epithelial CJ4.1 A cell lines [71]. These cell lines are generally easy to culture, and maintain crucial characteristics needed for drug permeability and transport, and disease modelling studies. However, immortalised cell lines are not always a true representation of the in vivo cell [72, 73]. Likewise, primary cells have also been widely used in ocular research. They have been used in investigating drug uptake and transport [74], barrier integrity [75], and pathology [76]. Ultimately, the choice between primary cells or immortalised cell lines comes down to the research question at hand, as both types have their own advantages and disadvantages.

### Advantages of 3D in vitro models in translational medical research

To recreate the complex ocular cellular make up and architecture in 2D in vitro models can be technically difficult, even with the development of techniques such as sandwich cultures, micropatterning, and adjusting substrate stiffness [77]. Therefore, advanced 3D cell culture models are more likely to accurately recapitulate this complexity compared to the 2D monolayer models.

By introducing a third dimension to conventional in vitro models, cells have increased cell-to-cell as well as cell-to-extracellular matrix (ECM) interactions [78]. The ECM is the structural component comprising all the proteins and polysaccharides surrounding the cells within the tissue. Although the same components are found in most ECM, the organisation and amount varies from organ to organ, and therefore makes ECM organ specific [79]. However, the ECM is not merely a structural support system, but also plays a role in cellular homeostasis, physiology, as well as disease progression [80]. Cells cultured in conventional 2D systems cannot fully recapitulate the complex 3D extracellular matrix architecture present in native tissue [81]. It is therefore critical for in vitro models to contain the appropriate ECM and cell-to-ECM contact to accurately recreate in vivo conditions.

These models also create nutrient, oxygen, and waste gradients, making them even more accurate models for in vivo conditions [25, 82]. Advanced 3D models are more appealing to use in drug kinetic and toxicological studies as they can recapitulate the drug distribution and delivery to cells observed in vivo [83, 84]. Most 3D techniques used can be divided into three categories: (i) Scaffold-free systems, (ii) Scaffold-based systems, and (iii) microphysiological systems.

**Fig. 2:**
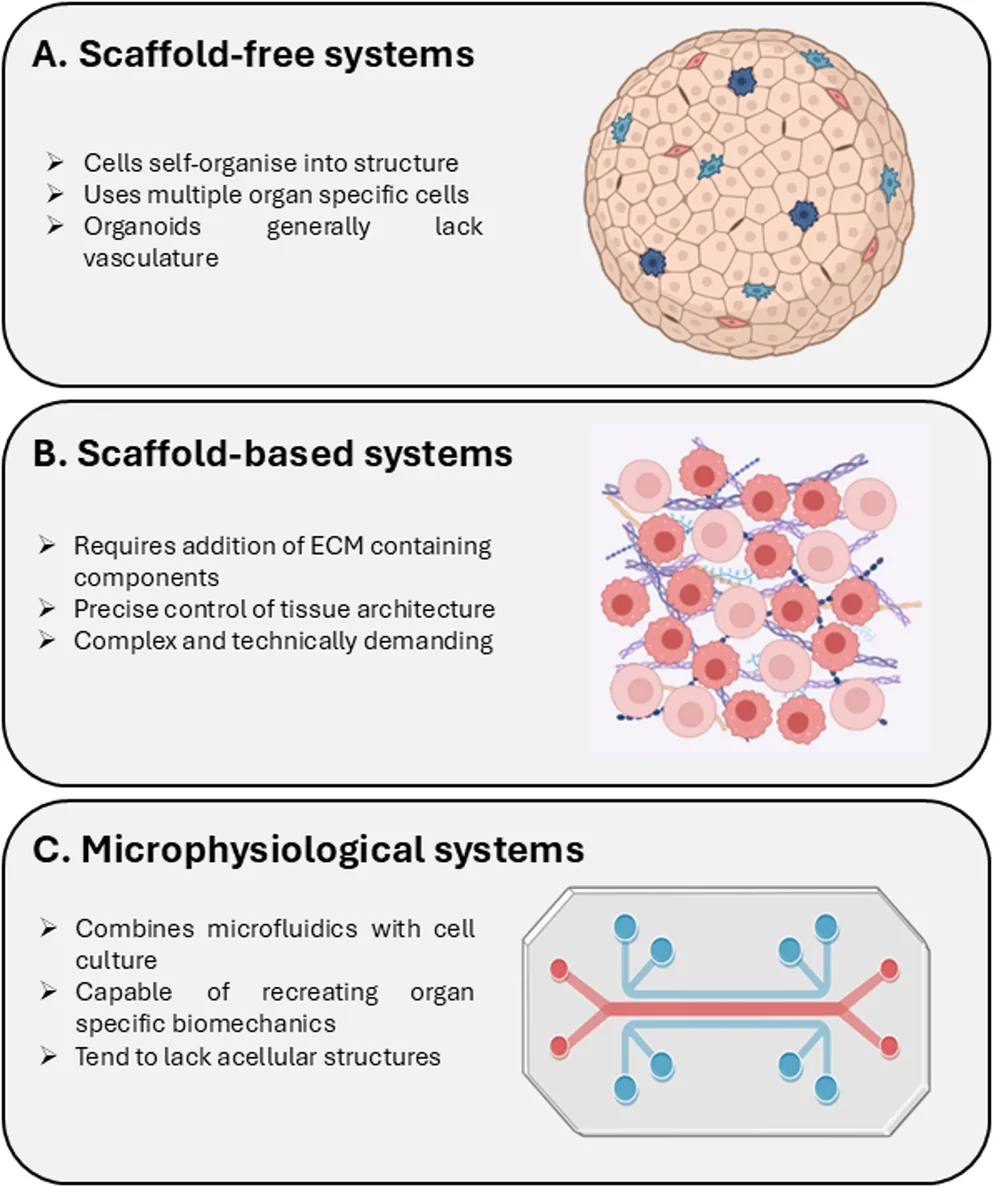
Three-dimensional in vitro models. (**A**) Scaffold-free systems like organoids are composed of multiple-cell types and can often self-organise into a 3D structure. (**B**)Scaffold-based systems such as hydrogels, bioprinting, and porous scaffolds provides ECM for cells to aggregate and proliferate into 3D constructs (**C**) Organ-on-a-chip technology is the most well-known microphysiological system and is often combined with microfluidics to create a more dynamic culture conditions as opposed to traditional static conditions

Scaffold-free systems or self-assembled systems, are systems capable of producing their own ECM without the need of external factors such as hydrogels, as previously reviewed [85]. Spheroids and organoids are the most popular examples of scaffold-free models. Organoids are an increasingly popular method to recapitulate in vivo conditions in vitro (Fig. 2A). Organoids are derived from ex vivo tissue and/or stem cells that self-organise into a structure which mimics the organ it is recreating, whilst consisting of organ specific cell types [86, 87]. These 3D structures can be cultured with two types of cells: (i) stem cells, and can be either embryonic stem cells (ESCs) or induced pluripotent stem cells (iPSCs) and (ii) primary isolated organ specific cells [88]. Their ability to mimic in vivo conditions makes them optimal to study ocular physiology and pathology. Organoids have great potential not only for investigating ocular physiology and pathology but also have potential clinical implications. The intrinsic cellular self-organisation of scaffold-free systems make them ideal to study developmental biology and disease mechanisms [89]. However, organoids lack standardisation and is still not capable of fully recapitulating complex architectures and microenvironments [90]. The creation of vascular organoids, although possible as shown by Ingaki et al. (2025) [91], is still complex, and a big limitation for the long term survival of organoids used in transplants [92].

On the other hand, scaffold-based systems relies on the use of biomaterials to provide the cells with structural support and a matrix to promote cellular attachment [93]. This category includes a wide range of models including the use of hydrogels, 3D bioprinting, and tissue engineering. Tissue engineering (TE) is the interdisciplinary application of biology and engineering principles to develop functional tissue or tissue-like substitutes with the use of cells, inert scaffolds, and bioactive factors (Fig. 2C) [94]. Recent advancements in the field of tissue engineering expanded on the techniques available in the field and now include biofabrication, bioprinting, bioink, and hydrogels [95]. Bioprinting, and other TE techniques, have two main advantages, the precise recapitulation of the macro-and micro-environment [96], and the ability to include non-cellular components such as ECM and growth factors [97]. This allows for the generation of vascularised tissue constructs [98], that can improve both pre-clinical as well as clinical research. However, hydrogels and bioink used within TE is often not compatible with cellular viability. Furthermore, due to the complex and interdisciplinary nature of TE, these models can also be quite technically challenging to work with [99]. Although bioprinting and hydrogels are often associated with TE, there are models that does not require these technical aspects of TE. Making use of the air-liquid interface (ALI) is one such method, by exposing the apical side of the cells to air and keeping the basal side submerged in media through a porous membrane, cells spontaneously form 3D tissue constructs [100]. By lifting cells to the ALI, cells stratify and differentiate [101] as well as promote the formation of tight junctions [102], creating a 3D construct. Scaffold-based approaches therefore provide improved control over tissue architecture and extracellular microenvironmental cues compared with scaffold-free systems, although the selection and optimisation of biomaterials remain critical because scaffold composition can directly influence cellular behaviour and tissue maturation [103].

The most well-known example of MPS is organ-on-a-chip (OOC) technologies. These are microdevices capable of recreating key characteristics of organs and tissues (Fig. 2B). These devices consist of a microfluidic chip that has been combined with a chamber that allows 3D cell growth. Organ-on-a-chip cannot only recapitulate key cellular architecture and function, but these models can also mimic the biomechanics of the organ of interest, such as blinking, contraction, or fluid sheer force [104]. Organ-on-a-chip technology, although still lacking for ocular models, have made great strides in recreating these biomechanical and unique architecture of the eye in vitro. However, more complex multi-cellular models are still limited, as well as the focus on acellular structures such as Bowman’s and Descemet’s membrane [105]. Despite these challenges, microphysiological systems offer a significant advantage over static culture systems by enabling controlled fluid flow and dynamic microenvironmental conditions, allowing more physiological relevant modelling of tissue function and cellular responses [106].

Scaffold-free, scaffold-based, and microphysiological systems all aim to recapitulate the 3D architecture of the native tissue, but each have distinct advantages and limitations.

Although all these models are often used in drug development, the recent advancements and development of these technologies also show great promise to be used in a clinical setting. These technologies now allow scientists to grow tissue constructs for translational research and even transplantation. The use of patient-derived organoids in autologous transplants is also a promising avenue in personalised medicine, as this can reduce the risk of immune rejection of the transplanted graft [107, 108].

However, 3D cultures are not without their limitations. One of the main challenges and limitations of these models is standardisation, with no standard protocol for the establishment and analysis of these models [109, 110]. Although these models are more advanced and improve on traditional 2D models, they are still not fully capable of reproducing entire physiological systems, mainly due to the immature nature of isolated cells [111]. Furthermore, these models can sometimes be overcomplicated and require more expertise than monolayer models [112], hindering the establishment of these models. Advanced models such as organ-on-a-chip requires additional equipment such as electronics, software, and microfluidic devices [113] which requires additional expertise and carries a higher cost to establishing these models.

### Advanced models for the anterior part of the eye

The anterior part of the eye is made up of numerous structures, but most models focus on the cornea. However, each cell type and tissue layer within the cornea has unique microenvironments and requirements to remain viable in vitro. These available models have been used in various applications (see Table 1 for summary) ranging from drug screening to clinical applications.

**Table 1:** Applications for 3D models of the anterior aspect of the eye

Technology	Tissue	Application	Key Findings	Ref
Organoid/ALI	Conjunctiva	Inflammation and clinical	• Maintained maturation markers • Infected with multiple viruses • Engrafted cells survived up to 3 weeks	[114]
OOC	Corneal Epithelium	Chemical Exposure	• Mimicked curvature of cornea and blinking • Dynamic exposure to chemicals	[115]
OOC/ALI	Corneal Epithelium	Epithelial integrity and infectious diseases	• Multi-layer (basement membrane and stromal layer) • Investigated epithelial integrity	[116]
ALI	Cornea	Infectious diseases and drug screening	• Commercially available • Key in vivo histological markers	[117]
Bioprinting	Corneal endothelium	Clinical	• One of few endothelial models • Bioprinting of cells for transplant	[118]
Hydrogel	Corneal epithelium	Clinical	• Hydrogel that lifts in non-degradable manner • Improves clinical outcome for transplant	[119]

The conjunctiva plays an important role in ocular inflammation [120]. Using human bulbar and palpebral conjunctiva, researchers successfully established conjunctival organoids from adult stem cells. Organoid formation was done by embedding the cells in a basement membrane extract (BME), an extracellular matrix hydrogel rich in basement membrane components such as laminin and collagen IV. Like tissue, these organoids expressed the epithelial marker KRT19 (also known as CK19) and epithelial progenitor marker TP63. Similarly, these organoids also expressed the keratinocyte differentiation markers MUC1 and AQP5 and later contained MUC5AC+ cells as well. To further promote epithelial maturation, organoid-derived cells were transferred to an ALI. These cells stratified within 4 days and had higher expression levels of MUC5CAC. They were also able to demonstrate the use of both models in studying conjunctivitis, by infecting the constructs with herpes simplex virus 1 (HSV1), human adenovirus 8, and severe acute respiratory syndrome coronavirus. The clinical implication of the organoids was demonstrated by engrafting organoids onto mechanically wounded conjunctiva of mice. They showcased the engraftment of the cells within 2 days post-transplant, and the survival of cells for up to 3 weeks [114]. This study examined the role of the conjunctiva, which is often under-represented, and further showed the potential application of organoids in a clinical setting for the anterior part of the eye.

The challenges commonly faced in ocular OOC systems have been addressed by Kim et al. (2025) where primary human keratocytes and corneal epithelial cells were cultured on a curved microporous scaffold designed to mimic the natural curvature of the cornea. Although this is a simplified representation of the corneal epithelium and stroma, they showed that the cells differentiated and stratified after being kept at the ALI for 7 days. Furthermore, the cells stained positive for the corneal epithelial markers CK3 and CK12, as well as the formation of a network of the tight junction proteins ZO-1 and occludin. The study also investigated the effect of various pollutants in solid, liquid, and gaseous states on the eye. By allowing liquid toxins to “wash” across the epithelial surface, rather than continuous submerged exposure, they mimicked the manner of exposure to these pollutants in a more physiological relevant manner than normal static in vitro models, which have potential implications for pharmaceutical studies for eye drops. Lastly, they established a blinking model by using soft polyester films that, with the help of an electromechanical mechanism, move from a saline reservoir, over the construct and back [115]. Although this study was focused on environmental pollutants, a curved, non-static, blinking-cornea on a chip model, has great implications for studies focused on ocular drug delivery and efficacy, by creating a much more representative model of the cornea.

Deng et al. (2024) showed the use of OOC technology by creating a “cornea-on-a-chip” system that included a basement membrane-mimicking porous membrane as well as a stromal layer composed of type I collagen containing primary human fibroblasts. By combining primary human corneal fibroblasts and immortalised HCECs under ALI conditions, the cells were well organised into a multi-layer tissue-like epithelium after 7 days. Furthermore, the cells also had an increase expression of the epithelial marker CK3, and the tight junction marker ZO-1. They further highlighted the potential of the OOC in studying epithelial integrity in pathologies like bacterial infection. This was demonstrated by introducing space-occupying structures within the growth chamber during culturing. Subsequently, the model was infected with Staphylococcus aureus to mimic bacterial keratitis and used to evaluate antibiotic efficacy. They highlighted the differential effect of commonly used antibiotics on both the stromal fibroblasts and epithelial cells [116]. This model combined various techniques to create a representative model. By creating a tissue layer, it is possible to investigate epithelial integrity in a more reliable manner than a traditional 2D monolayer.

A commercially available 3D human corneal epithelium equivalent (HCEE), generated through ALI exposure, has been used to investigate various viral infections. This construct, called SkinEthic™ HCE, consists of immortalised human corneal keratinocytes and forms a multilayered epithelium of 5–6 cell layers. Both cuboidal and columnar cells are found in the basal layer, with squamous cells at the apical surface, making it histologically similar to native corneal epithelium [117]. This construct was used to study the progression of herpes stromal keratitis by exposing the construct to HSV1, varicella zoster virus (VZV), and human cytomegalovirus (hCMV). Not only was this commercial construct able to show differences in infection patterns, such as HSV1 and hCMV infecting intact tissue while VZV required epithelial damage for infection. They further showed the drug screening potential of this construct by testing the effect of the antiviral drug acyclovir, which significantly reduced HSV1 infection in the model. The importance of 3D models was shown with this study by showing that infectious diseases and treatments affect different layers of the same tissue.

With regards to bioprinting the choice of the correct bioink is crucial, for both cell support as well as the application of the bioprinted tissue. This was shown by Grönroos et al. (2024) and his team, who investigated the optimal bioink for printing human pluripotent stem cell-derived corneal endothelial cells for transplantation [118]. They made use of a hyaluronic acid-based hydrazone crosslinked ink that was supplemented with collagen IV and laminin 521 and improved the cytocompatibility of the ink. The viability of the cells was tested by printing the cells onto ex vivo rat, porcine, and human Descemet’s membrane which had the endothelial layer removed. The printed endothelial cells did not migrate into the stroma and maintained the expression of key endothelial markers (ZO-1, Na^+^/K^+^-ATPase and CD166) in both the rat and porcine model. Cells printed onto the human Descemet’s were smaller and had a higher density compared to cells without the ink, whilst the bioink also allowed for precise spatial control of cells during printing. This highlights the importance of optimising an appropriate bioink-cell interaction and showcases the potential of bioprinting in the clinical setting.

With the emergence of hydrogels and bioinks for use in clinical applications, detachment methods for these constructs from supporting structures, while maintaining construct integrity and cellular viability, are crucial. An enzymatic approach is often followed. However, during this process enzymes can cleave proteins on the cell surface and compromise the construct integrity [121, 122]. For this reason Tsurkan et al. (2025) created a hydrogel that can be selectively degraded in a controlled manner, preserving tissue and ECM integrity, ultimately improving the clinical implications for tissue constructs [119]. They introduced a small peptide motif within their gel cleavable by the common blood coagulation factor FXa. The hydrogel served as both a structural support for cultured immortalised HCEC-B4G12 cells and as a matrix mimicking the stroma and Descemet membrane of the cornea. The adherent cells showed the expression of ZO-1 and the formation of cell sheets with the main components of endothelial extracellular matrix proteins. Tissue constructs lifted with the FXa showed no alteration in their metabolic state, and shrank up to 60% upon lifting, which indicated that tight junctions remained intact after lifting, and was confirmed with ICC. The lifted construct was transplanted onto a de-endothelialised ex vivo porcine cornea and showed that the hydrogel adapted to the curvature of the cornea and attached to the Descemet’s membrane as a confluent endothelial monolayer.

### Models for the posterior part of the eye

While the posterior part of the eye contains fewer structures compare to the anterior part, majority of ocular research on advanced in vitro models have focused on this region, specifically the retina (see Table 2).

**Table 2:** Applications for 3D models of the posterior aspect of the eye

Technology	Tissue	Application	Key Findings	Ref
Organoid	Retina	Pathophysiology	• Investigated genetic mutation • ROs formed from patients • Displayed in vivo pathophysiology	[123]
Organoid	Retina	Physiology	• ROs responded to light • Key in vivo architecture	[124]
Organoid	Retina	Transplant	• Effect of immunosuppressants on RO • Long term follow up on grafts	[125]
Organoid	Retina	Transplant	• Isolated retinal ganglion cells from RO • Survived in mice for up to 3 weeks	[126]
Organoid	Retina	Transplant	• Few human clinical trial • Long term follow up • ROs not induced adverse effects	[127]
OOC	Blood-retinal-barrier	(Patho)-physiology	• One of few non-organoid models • Showcased key role of microvasculature • Investigated diabetic retinopathy	[128]

Retinal organoids (RO) created by Han et al. (2024), were used to study X-linked juvenile retinoschisis (XLRS), which is caused by a mutation in the retinoschisis (RS1) gene. These organoids were formed using human iPSC (hiPSCs) derived from peripheral blood mononuclear cells (PBMCs) collected from patients with RS1 mutations. The ROs were allowed to differentiate and showed the expression of the retinal progenitor markers CHX10 and PAX6 at days 30–40 of differentiation, with the RS1 mutated ROs showing no differences compared to ROs isolated from healthy control individuals. They were able to show that ROs from XLRS patients had decreased Na/^+^K^+^-ATPase subunits (ATP1A3 and ATP1B2), as well as increased mitogen-activated protein kinase (MAPK) signalling, with increased p-ERK1/2, and lowered retinal cell marker levels [123]. This study highlights the important role that patient derived organoids (PDOs) have in personalised medicine. By using patient-derived cells, key genetic differences can be recreated in vitro that can greatly aid in the clinical outcome of these patients.

Organoids can also be used to better understand cellular and tissue function at a molecular level, and in the case of ROs, can help us understand the role of rods and cones as shown by Saha et al. (2022). Here, Saha and colleagues showed that mature ROs between 240 and 270 days can elicit responses to different light stimuli through cone photoreceptors as shown by patch-clamp electrophysiology recordings and were capable of reproducing this across ROs from multiple hiPSC sources. These organoids had an outer segment cone photoreceptor region capable of responding to both medium and long wavelengths with wavelength-specific light responses characteristics of cone phototransduction. Furthermore, they also showed that the ROs contained a rod : cone ratio of 4 : 1, which is comparable to the parafovea of primates [124]. Although only approximately 35% of cones were light sensitive, and they could not detect a measurable response in the rod photoreceptors, this study shows promising results for study molecular pathways, and conditions like colour blindness.

Sims et al. (2025) utilised human ESCs (hESCs) in xeno-free conditions to form ROs. These organoids displayed no expression of the pluripotency marker OCT4, whilst approximately 75–80% of the organoids tested positive for retinal maturation markers OTX2 and RAX after 55 days. Through co-culturing the ROs with immune cells using mixed lymphocyte reactions the authors demonstrated that ROs exhibit low immunogenicity and induce minimal immune activation in vitro, suggesting that immunosuppressive drugs are unlikely to negatively affect RO development or metabolism during transplantation. Lastly, these ROs were transplanted into both male and female rats and the graft sites were imaged 2 weeks, 1 month, and 2 months post-transplantation to show the stability of the grafts [125]. This long-term follow up on graft stability demonstrated that ROs are not affected by immunosuppressants, which is a problem faced in solid organ transplantation [129].

Similarly, Li et al. (2024) showed the potential of ROs to grow retinal ganglion cells (RGC) that can be used for replacement therapies in diseases like glaucoma [126]. They were able to successfully isolate RGC from 40–60-day-old retinal organoids using FACS sorting of CD184^+^CD171^+^ cells, which expressed RGC markers such as Brn3b and βIII-tubulin, and transplant it into mice with injured optic nerves. These cells were able to survive in the mice for up to 3 weeks and showed integration toward the damaged optic nerve region.

Focused more on the long-term survival of organoid derived tissue, a team from Japan transplanted retinal organoid sheets into two patients with retinitis pigmentosa and monitored the patients and grafts for 2 years. The graft thickness continued to increase with time and showed no signs of overgrowth until 104 weeks. Neither inflammation nor retinal vascular pathology was observed in either patient during this period. Although no improvement in their visual acuity was observed, this study shows the safe use of organoid tissue in transplantation [127]. Importantly, this study demonstrates that ROs can adapt and grow within a host environment, which highlights the potential clinical use of these ROs as “mini-organs” for transplantation purposes.

By incorporating controlled microfluidics within OOC technology, it is possible to investigate cellular responses towards controlled fluid flow, such as in microvessels and the inner blood-retinal barrier (iBRB). This was shown by a team that created a perfusable iBRB on-a-chip, using human retinal microvascular endothelial cells (HRMVECs), retinal pericytes, and retinal astrocytes embedded in a fibrin ECM within a microfluidic device. These cells self-organised into microvascular networks with similar an architecture as the in vivo iBRB, with a perfusable lumen. The resulting microvascular networks expressed key barrier markers including CLDN5, ZO-1, and VE-cadherin and produced basement membrane components such as laminin and collagen IV, confirming the formation of a functional barrier. They also modelled diabetic retinopathies by perfusing the microvascular networks with high glucose (30mM) together with the pro-inflammatory cytokines TNF-α and IL-6. Under these diabetic conditions, the pathological networks decreased to approximately 30% of the size of the control networks, with a reduced perfusability and loss of pericyte coverage and formation of ghost vessels also observed [128]. These phenotypes replicate early microvascular changes associated with non-proliferative diabetic retinopathy, demonstrating that the model can be used to study disease mechanisms and evaluate therapeutic strategies targeting the microvasculature.

## Conclusion

The three-dimensional cell culture models reviewed in this manuscript, i.e. organoids, organ-on-a-chip, and tissue engineered constructs, can recapitulate the in vivo environment more accurately than traditional monolayer models. Recent advances within advanced ocular cell culture models have started to address ocular specific challenges such as vascularisation within these constructs, curvature of the cornea, blinking, and the tear film. These aspects are crucial in models to study diseases and in drug development, but it is also an important step towards creating “mini-organs” for clinical applications such as transplants. The current shift seen in advanced ocular in vitro models towards more clinical based applications can notably improve patient outcomes in transplants, when combine with a personalised medicine approach.

Despite these advances, several challenges still limit the widespread adoption of advance in vitro ocular models as the standard platforms for disease modelling and drug discovery. Many of these systems remain technically complex and require specialised expertise and equipment, which can limit reproducibility and standardisation. In addition, regulatory validation frameworks for drug development are still largely based on established animal models, making the integration of novel in vitro platforms into preclinical pipelines more challenging. These factors currently limit the broader implementation of advanced ocular models in both research and pharmaceutical development.

Furthermore, studies would need to investigate the long-term viability of engineered grafts and “mini-organs” used in transplant to fully understand the clinical use of these constructs. This involves investigating the viability timeframe of these constructs in vivo as well as the interactions with the native in vivo tissue. Furthermore, very few studies have focused on factors such as corneal transparency or ocular pressure. These are crucial aspects for both pre-clinical applications, drug discovery and disease modelling, as well as clinical applications. Overall, advanced ocular in vitro models hold enormous potential for drug discovery, disease modelling, and clinical applications, with ongoing research expected to significantly shape the future of ocular research and therapeutic development.

## Data Availability

No datasets were generated or analysed during the current study.

## Abbreviations

2D: Two dimensional
3D: Three dimensional
ALI: Air-liquid interface
BME: Basement membrane extract
ECM: Extra cellular matrix
ESCs: Embryonic stem cells
HCEE: Human corneal epithelium equivalent
hCMV: Human cytomegalovirus
hiPSCs: Human induced pluripotent stem cells
HSV1: Herpes simplex virus 1
iPSCs: Induced pluripotent stem cells
LCA: Leber’s congenital amaurosis
LSCD: Limbal stem cell deficiency
MAPK: Mitogen activated protein kinase
MPS: Microphysiological systems
NAMs: New Approach Methodologies
OOC: Organ-on-a-chip
PBMCs: Peripheral blood mononuclear cells
PDOs: Patient derived organoids
RO: Retinal organoid
RPE: Retinal pigment epithelium
SJS: Stevens Johnson Syndrome
TE: Tissue engineering
TEN: Toxic epidermal necrolysis
VZV: Varicella zoster virus
XLRS: X-linked juvenile retinoschisis
